# Primary Tumor Resection Decelerates Disease Progression in an Orthotopic Mouse Model of Metastatic Prostate Cancer

**DOI:** 10.3390/cancers14030737

**Published:** 2022-01-31

**Authors:** Johannes Linxweiler, Turkan Hajili, Philip Zeuschner, Michael D. Menger, Michael Stöckle, Kerstin Junker, Matthias Saar

**Affiliations:** 1 Department of Urology, Saarland University, 66421 Homburg, Saar, Germany; hajilitu@diako.de (T.H.); philip.zeuschner@uks.eu (P.Z.); michael.Stoeckle@uks.eu (M.S.); kerstin.Junker@uks.eu (K.J.); msaar@ukaachen.de (M.S.); 2 Institute for Clinical-Experimental Surgery, Saarland University, 66421 Homburg, Saar, Germany; michael.menger@uks.eu

**Keywords:** cytoreductive primary tumor removal, metastases, orthotopic mouse model, oligometastatic prostate cancer, radical prostatectomy

## Abstract

**Simple Summary:**

In this preclinical in-vivo study, we used an orthotopic prostate cancer mouse model to analyze the effects of primary tumor resection on further disease progression and survival in metastatic prostate cancer. Sixty-four mice with metastatic prostate cancer, induced by intraprostatic injection of three-dimensional prostate cancer spheroids, were randomized into two groups: one group received resection of their primary tumor while the other group received a sham operation. After this, the mice were followed-up for 10 weeks. In comparison with the sham operation group, mice with primary tumor resection showed significantly slower PSA progression, less lung metastases, and significantly longer survival. These results are a hint towards a beneficial oncological effect of primary tumor resection in metastatic prostate cancer. Furthermore, the established versatile in-vivo model can be used to study the molecular mechanisms of primary tumor/metastasis interaction in prostate cancer.

**Abstract:**

Radical prostatectomy in oligometastatic prostate cancer is a matter of intense debate. Besides avoiding local complications, it is hypothesized that primary tumor resection may result in better oncological outcomes. The aim of our study was to analyze the effect of primary tumor resection on disease progression in an orthotopic prostate cancer mouse model. First, the optimal time point for primary tumor resection, when metastases have already occurred, but the primary tumor is still resectable, was determined as 8 weeks after inoculation of 5 × 10^5^ LuCaP136 cells. In a second in vivo experiment, 64 mice with metastatic prostate cancer were randomized into two groups, primary tumor resection or sham operation, and disease progression was followed up for 10 weeks. The technique of orthotopic primary tumor resection was successfully established. Compared with the sham operation group, mice with primary tumor resection showed a significantly longer survival (*p* < 0.001), a significantly slower PSA increase (*p* < 0.01), and a lower number of lung metastases (*p* = 0.073). In conclusion, primary tumor resection resulted in slower disease progression and longer survival in an orthotopic mouse model of metastatic prostate cancer. In future studies, this model will be used to unravel the molecular mechanisms of primary tumor/metastasis interaction in prostate cancer.

## 1. Introduction

The possible beneficial effects of treating prostate cancer (PCa) via radical prostatectomy (RP) in patients suffering from metastatic PCa are a matter of controversial debate in recent years [1,2]. While cytoreductive primary tumor resection is well established in other cancers [3,4,5], this concept has only recently been included in the scientific debate over PCa. In addition to avoiding local complications, another rationale for RP in patients with metastasized PCa is to slow down disease progression by inhibiting the interaction between the primary tumor and metastases and removing potentially lethal tumor cell clones [6,7,8]. Several retrospective studies have shown that disease progression can be influenced by RP [9,10,11], and primary tumor treatment in oligometastatic PCa proved beneficial in the STAMPEDE trial [12]. Furthermore, primary tumor treatment may be associated with a better and longer-lasting response to androgen deprivation therapy (ADT), and RP of locally advanced tumors after inductive ADT can yield promising results [13,14]. However, cytoreductive RP is being tested in ongoing prospective trials whose results are still pending [15,16,17]. Since novel imaging modalities based on prostate-specific membrane antigen (PSMA) are entering clinical routine, it is becoming evident that most of the high-risk localized PCa patients treated by RP or radiotherapy plus ADT do not, in fact, have localized but rather oligometastatic PCa [18,19]. Therefore, the number of patients suffering from oligometastatic PCa is probably much higher than previously thought, and a better understanding of the biology of this disease stage and a selection of optimal treatment approaches are necessary.

It is of crucial importance to establish valid preclinical models of metastatic prostate cancer to understand the biological mechanisms underlying local tumor progression, the shift from organ-confined to oligometastatic to polymetastatic disease, the interaction between the primary tumor and metastases, and the effects of interventions such as metastasis-directed therapy (MDT) or primary tumor treatment.

Our group has considerable expertise in developing innovative orthotopic PCa mouse models [20,21,22,23]. Previously, we created a model based on the intraprostatic injection of 3D LuCaP136 PCa spheroids, which leads to the growth of locally invasive, androgen receptor (AR)-positive and prostate-specific antigen (PSA)-producing tumors and the development of lymph node and lung metastases [22,23].

This study established a technique of primary tumor resection in a representative orthotopic mouse model of metastatic prostate cancer and analyzed the effects of primary tumor resection on disease progression.

## 2. Materials and Methods

### 2.1. Cell Culture

LuCaP136 spheroids were cultured at 37 °C in ultra-low-attachment plates (Corning Inc., Corning, NY, USA) in optimized StemPro stem cell medium (Thermo Fisher Scientific, Waltham, MA, USA) and processed for orthotopic injection, as previously described [21,22]. LuCaP136 is a three-dimensional spheroid culture established from the LuCaP136 patient-derived xenograft (PDX) model [21,24], which was generated using tumor cells from the ascites of a metastatic castration-resistant prostate cancer patient (obtained via a rapid autopy program in Seattle) [25]. This patient was 62 years old and had received androgen deprivation therapy (ADT) but no chemotherapy. Molecular and biologic features of this spheroid cell line and the respective xenograft inculde the expression of a wild-type androgen receptor (AR), expression of the prostate-specific antigen (PSA), loss of both *PTEN* alleles, absence of the *TMPRSS2:ERG* fusion gene, good response to castration, and the development of osteosclerotic lesions when implanted into the bone of immunodeficient mice [21,24,25].

### 2.2. Mice

Male, 8–10-week-old SCID (severe combined immunodeficiency) mice (CB17/lcr-*Prkdc^scid^*/lcrlcoCrl) were obtained from Charles River Laboratories (Sulzfeld, Germany). The mice were kept in isolated ventilated cages under specific-pathogen-free conditions in a temperature- and humidity-controlled 12 h/12 h dark/light environment at the animal care facility of the Institute for Clinical-Experimental Surgery, Saarland University, Germany. They had free access to tap water and standard pellet food.

All animal experiments were approved by the local governmental animal care committee (No. 30/2015 and 26/2020) and conducted in accordance with the German legislation on animal protection.

### 2.3. Intraprostatic Tumor Cell Inoculation

**Figure 1:**
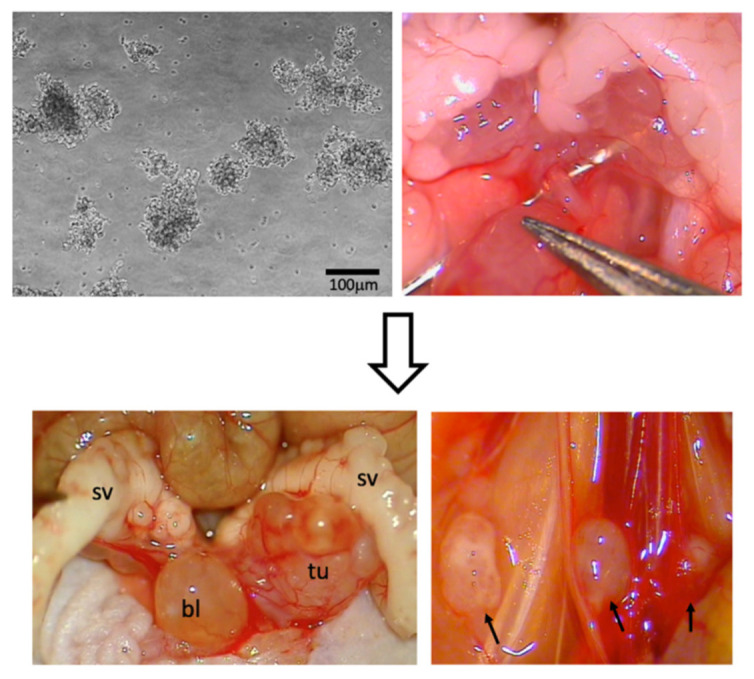
Orthotopic mouse model of oligometastatic prostate cancer. Injection of 3D LuCaP136 spheroids into the left anterior prostate lobe of CB17/lcr-*Prkdc^scid^*/lcrlcoCrl SCID mice (upper row) results in the development of a locally invasive, intraprostatic primary tumor and multiple lymph node metastases (lower row; metastases marked with arrows). bl = urinary bladder, sv = seminal vesicles, tu = tumor.

For orthotopic inoculation of LuCaP136 spheroids, the mice were anesthetized intraperitoneally (75 mg/kg ketamine, 15 mg/kg xylazine). Intraprostatic tumor cell inoculation was performed under a stereo-microscope (Leica M651; Leica Microsystems AG, Heerbrugg, Switzerland), as previously described (22). Briefly, 10 μL of a 1:1 Matrigel:StemPro suspension containing 5 × 10^5^ LuCaP136 spheroids was injected into the left anterior prostate lobe of the mice using a cooled 10-μL Hamilton syringe (VWR International, Darmstadt, Germany) (Figure 1).

**Figure 2:**
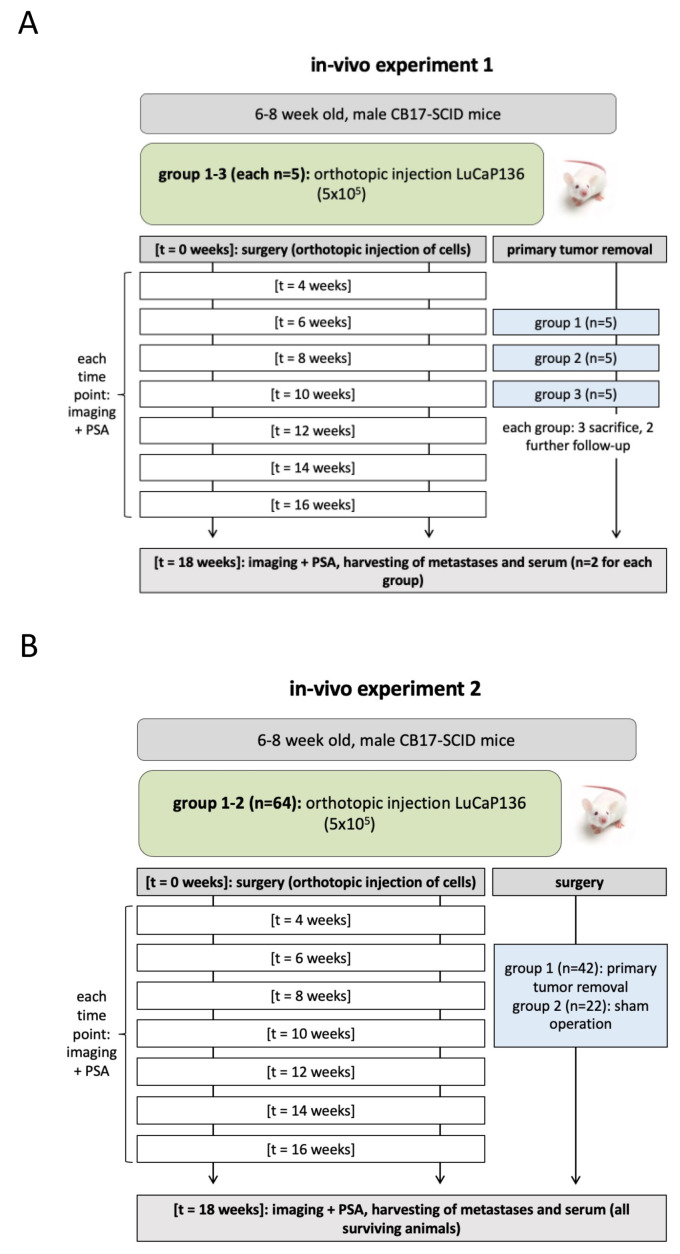
Schematic illustration of in vivo experiments. In experiment 1 (**A**), the optimal time point for cytoreductive primary tumor resection was determined. In experiment 2 (**B**), mice were randomized into a group that underwent primary tumor resection and a group that underwent a sham operation. The mice were further followed up to analyze the effect of primary tumor resection on disease progression and survival. PSA = prostate-specific antigen.

The study approach consisted of two consecutive in vivo experiments (Figure 2). Experiment 1 (Figure 2A) identified the optimal time point for cytoreductive primary tumor resection, i.e., a time point when all mice have lymph node metastases, but the primary tumor is still resectable. Therefore, LuCaP136 spheroids were orthotopically inoculated in 15 mice (3 groups with 5 mice each). In each group, at week 6, 8 or 10 after orthotopic tumor cell inoculation, 3 mice were sacrificed and their lumbar aortic lymph nodes excised to look for metastases, while the other 2 mice underwent primary tumor resection and further follow-up.

In experiment 2 (Figure 2B), 64 mice received intraprostatic LuCaP136 spheroid injection. Of these, 42 mice were randomized into a primary tumor resection group and 22 to a sham operation group and their disease burden was monitored following these interventions for 10 weeks.

### 2.4. Monitoring of Disease Burden

Intraprostatic injection of LuCaP136 spheroids led to the development of locally invasive tumors and lymph node and lung metastases (Figure 1) [22,23]. Local and systemic tumor growth were monitored using serum PSA measurement and high-resolution 3D ultrasonography every 2 weeks, beginning from week 4 after tumor cell inoculation, as previously described [20,23]. Furthermore, a contrast-enhanced in vivo micro CT of all animals was performed at week 12 after tumor cell inoculation. Blood sampling during the study was performed by puncture of the retrobulbar venous plexus at weeks 4 to 16 and by puncture of the inferior vena cava during at week 18.

### 2.5. Lymph Node Dissection

For lumbar aortic lymph node dissection, the mice were anesthetized intraperitoneally (75 mg/kg ketamine; 15 mg/kg xylazine). They were placed in a supine position, and the skin was opened by a 10 mm lower abdominal midline incision under stereo-microscopic control (Leica M651; Leica Microsystems AG, Heerbrugg, Switzerland). After laparotomy, the bladder, seminal vesicles, and anterior prostate lobes were mobilized and exposed through the incision. Lumbar aortic lymph nodes were identified in their typical location [26], carefully excised, fixed in formalin, embedded in paraffin, stained with H&E using a standard protocol, and examined for the presence of cancer cells. The mice were euthanized after this intervention (part of in vivo experiment 1).

### 2.6. Primary Tumor Resection/Sham Operation

For cytoreductive primary tumor resection, intraperitoneal anesthesia and opening of the abdomen were performed as described above. The intraprostatic tumor in the left anterior prostate lobe was identified and completely excised using spring-loaded scissors (Fine Science Tools GmbH, Heidelberg, Germany), carefully avoiding injury to the left seminal vesicle, the left ureter, and the urinary bladder. Hemostatic control was achieved using a combination of thermocoagulation (Geiger Thermal Cautery Unit; Geiger Medical Technologies, Council Bluffs, IA, USA) and Tabotamp hemostatic agents (Ethicon, Somerville, NJ, USA). Finally, the incision was closed in two layers.

For the sham operation group, intraperitoneal anesthesia and opening of the abdomen were performed, as described above. Then, the incision was closed in two layers without any further steps.

The number of mice randomized into the primary tumor resection group was much higher than that of the sham operation group since we expected a higher rate of loss in the former group due to higher surgical trauma.

### 2.7. Statistics

Statistical analyses were performed using Microsoft Excel for Mac V16.46 (Microsoft Corporation, Redmond, WA, USA), SigmaPlot version 13 (Systat Software Inc., San Jose, CA, USA), and SPSS Statistics 23 (IBM, Armonk, NY, USA). Two-tailed statistical tests were performed, and *p* < 0.05 was considered statistically significant. The statistical tests used in individual settings are stated in the respective sections of the main text and figure captions.

## 3. Results

**Figure 3:**
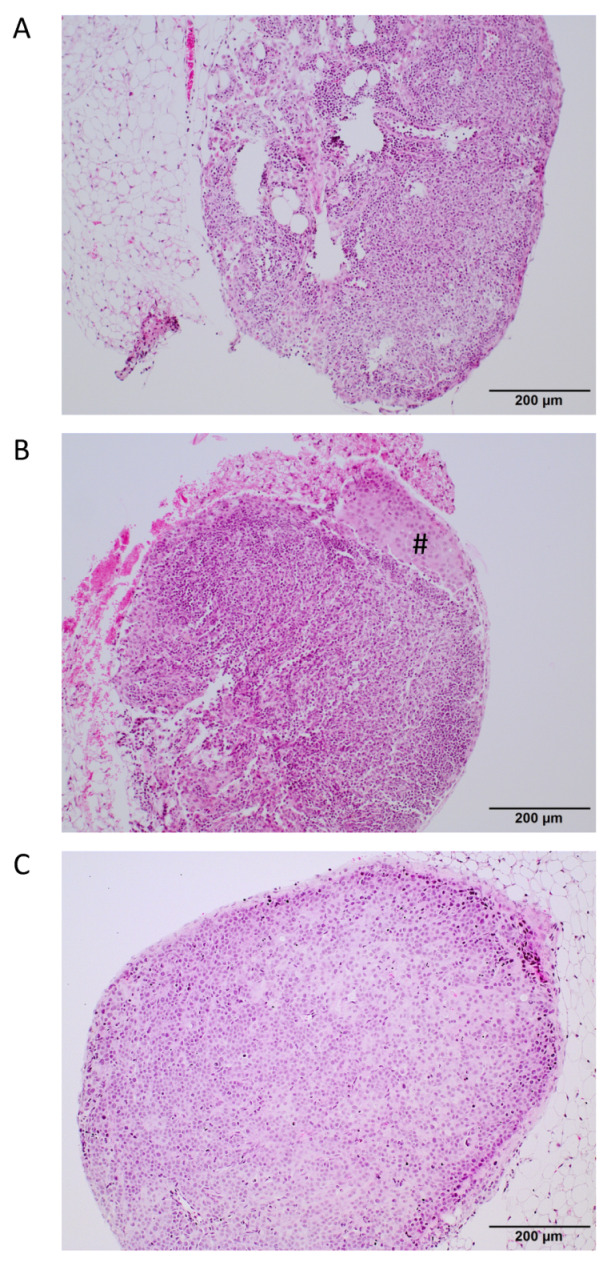
Histological analysis of lymph nodes for the presence of metastases. Representative H&E-stained sections of a tumor-free lymph node (**A**), a lymph node bearing a small tumor cell infiltrate (marked with #) (**B**); tumor cells marked with a rhomb, and a lymph node completely occupied by tumor cells (**C**). Scale bar = 200 μm.

In experiment 1, the primary tumor proved to be technically resectable at weeks 6 and 8 but not at week 10. Resectability was defined as the possibility to completely or almost completely resect the primary tumor without injuring the urinary bladder, the rectum, or the ureter. All mice had histologically proven lymph node metastases from week 8 onward (Figure 3). Lung or other distant metastases were not present at this time point. Therefore, week 8 after orthotopic tumor cell inoculation was identified as the optimal time point for primary tumor resection (Table 1).

**Figure 4:**
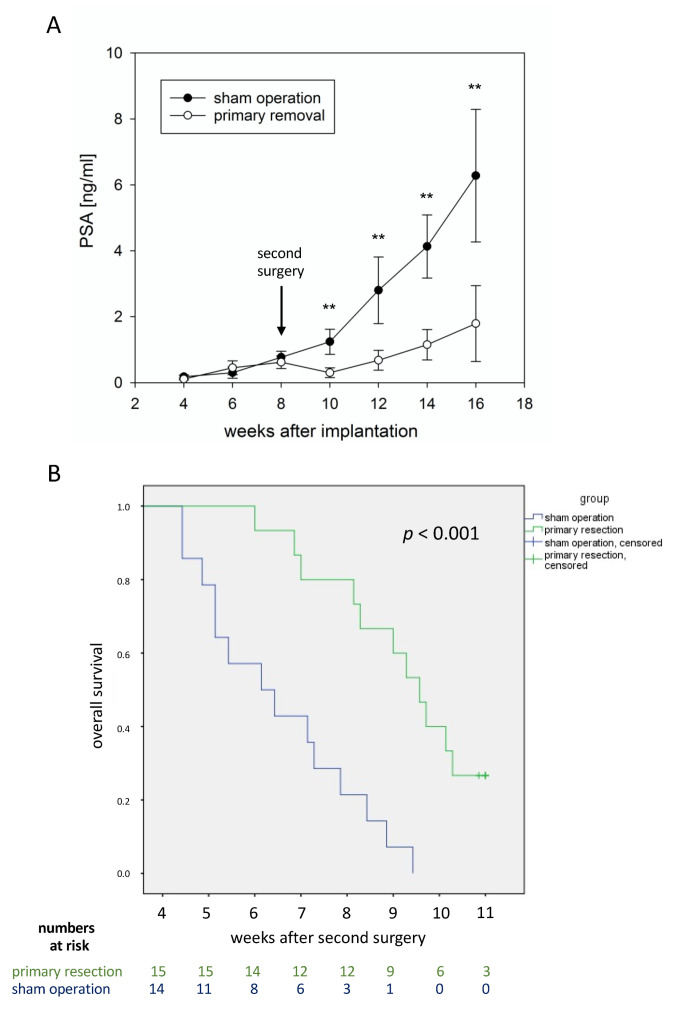
PSA progression and survival after cytoreductive primary tumor resection. (**A**) Development of PSA values in the serum of mice (retrieved by puncture of the retrobulbar venous plexus) after sham operation (black dots) and after primary tumor resection (white dots). The medians and standard deviations for all mice still alive at the respective time point in each group are shown. (**B**) Kaplan–Meier curves illustrating the survival of mice after sham operation (blue curve) or primary tumor resection (green). Of note, the last surviving animals were sacrificed a few days later than week 18, thus explaining the presence of the time point “11 weeks” in this Kaplan-Meier curve. The *p*-value in the graph was calculated using a log-rank test. ** *p* < 0.01.

**Figure 5:**
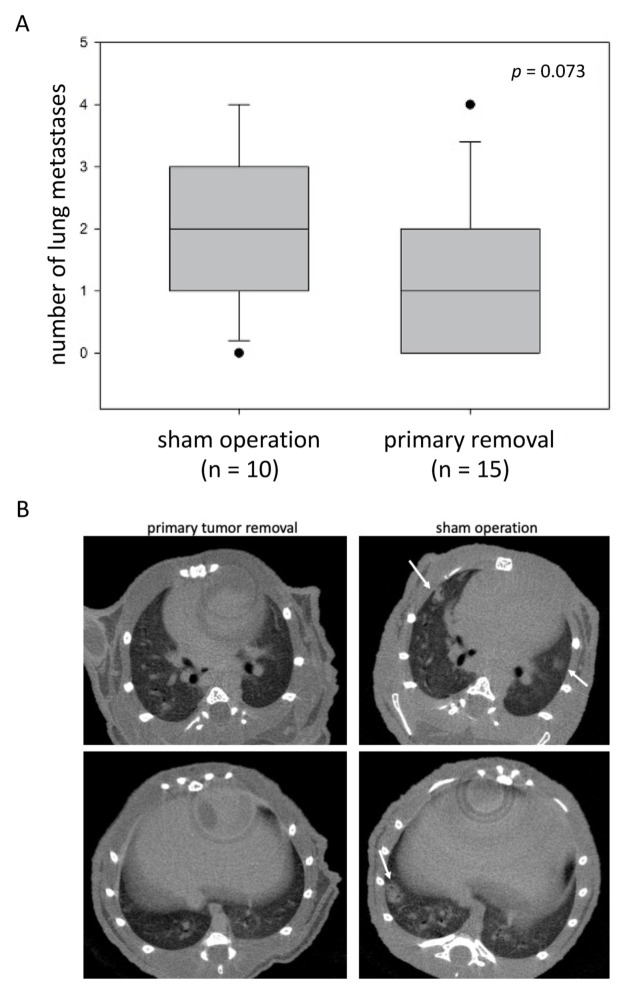
Extent of lung metastases after sham operation or primary tumor resection. (**A**) Number of lung metastases after sham operation or primary tumor resection, as determined by high-resolution in vivo micro-CT at week 12 after orthotopic tumor cell inoculation. (**B**) Representative cross-sectional micro-CT images of a mouse without lung metastases (after primary tumor resection, left column) and a mouse with multiple lung metastases (after sham operation, right column, metastases marked with an arrow).

In experiment 2, 5 of the 64 mice did not survive the first surgery (tumor cell inoculation). All remaining 59 mice underwent the second surgery at week 8 (39 mice in the primary tumor resection group and 20 in the sham operation group). Unfortunately, 30 of these 59 mice died during the second surgery or in the first two weeks after the second surgery (24 of 39 from the primary tumor resection group and 6 of 20 mice from the sham operation group; *p* = 0.02 for intergroup comparison). These deaths were considered to be associated with surgery/with surgical stress and not with tumor progression, which was confirmed by autopsy in all cases. Therefore, the remaining 29 mice were included in further oncological follow-up (15 in the primary tumor resection group and 14 in the sham operation group). The primary tumor resection group showed significantly slower PSA increase (Figure 4A; *p* < 0.01) and significantly longer survival (Figure 4B; *p* < 0.001) compared with the sham operation group. Furthermore, there was a trend towards less lung metastases in the primary tumor resection group compared with the sham operation group (median number of 1 vs. 2 lung metastases, respectively; *p* = 0.073; Figure 5).

## 4. Discussion

This study applied an innovative orthotopic mouse model to study the effects of primary tumor resection in oligometastatic prostate cancer. A beneficial effect of this intervention on disease progression was demonstrated by means of significantly slower PSA increase, significantly longer survival, and less lung metastases after primary tumor resection compared with the sham operation. To exclude deaths probably due to surgical trauma and not to tumor progression, only mice that survived the second surgery for more than two weeks were included in oncological outcome analyses. Although a considerable number of mice were excluded from this final analysis, significant results were obtained, strongly suggesting real beneficial effects of primary tumor resection. Regarding PSA kinetics, it is expected that PSA levels reduce in mice after primary tumor resection due to reduced tumor mass; however, the slope of the PSA curve was less steep after primary tumor resection than after sham operation, indicating slower progression of residual tumor metastases.

Our results support data from two other studies: one study reporting the effect of primary tumor resection on disease progression in an orthotopic PCa mouse model [27] and the other describing a RP technique in mice [28].

Cifuentes et al. provided, for the first time, preclinical in vivo data suggesting a beneficial effect of primary tumor resection in metastasized PCa [27]. In this study, orthotopic xenografts were grown in ten NOD-SCID mice via intraprostatic injection of PC3 cells, and 30 days after tumor cell inoculation, the primary tumor was resected in half of the mice, while the remaining mice were not treated. The primary tumors were not completely removed, since macroscopically visible local recurrences were observed at autopsy. After 70 days of follow-up, the treatment group showed fewer and smaller metastases by bioluminescence and autopsy compared with the control group. Di Trapani et al. presented the first mouse model of radical prostatectomy [28]. They performed complete resection of the prostate in eight C57BL/6 wild-type mice and eight PCa-bearing TRAMP mice. At the end of surgery, a permanent vesicocutaneous fistula was constructed. All mice survived for the first few postoperative days, and 14 of 16 mice were still alive three months postoperatively.

Both studies successfully applied complex surgical techniques and obtained promising results; however, they also faced methodological constraints, limiting their validity. In both studies, artificial in vivo PCa models were used. In the genetically engineered TRAMP mouse model, SV40 antigen expression in prostatic epithelial cells induced the development of intraprostatic adenocarcinomas [29]. However, the SV40 antigen is an oncogene originating from a polyomavirus, which is normally not present in human cells. In addition, the overexpression of a single antigen does not adequately represent the molecular complexity and heterogeneity of human PCa. PC3 cells are of human origin, but it is doubtful that they can represent human PCa, considering a lack of androgen receptor and PSA expression [30]. Di Trapani et al. [28] did not perform oncological follow-up of the mice but monitored their health status for the first 3 months postoperatively. Cifuentes et al. used bioluminescence imaging for non-invasive monitoring of the tumor burden after primary tumor resection [27], which is a sensitive method of detecting metastases; however, metastases can only be quantified roughly, at least when 2D image acquisition is used.

We can only speculate on the possible biological mechanisms that might contribute to observed beneficial effects of primary tumor resection in our model [20,31,32]. On the one hand, the primary tumor may be a beehive-like source of continuous tumor cell seeding leading to the formation of new metastases. On the other hand, the primary tumor could foster metastasic dissemination by the secretion of soluble factors—like growth factors, chemokines, or extracellular vesicles which contribute to the formation of premetastatic niches and/or stimulate the growth of metastases. However, this will have to be further elucidated in future experiments focusing on these questions. Here, our model established in this study will serve as a valuable experimental tool.

The strengths of our study include the use of an innovative and close-to-reality in vivo model by orthotopically xenografting 3D PCa spheroids, which show many molecular traits typical for PCa [21,22]. Furthermore, small-animal imaging with high-resolution ultrasonography and in vivo micro-CT as well as serum PSA measurements are highly reliable and validated methods [22,23] of tracking the disease burden in these mice. For future studies, we aim to further use bioluminescence as an even more sensitive tool to detect metastases in our orthotopic model; however, this method was not yet available for the experiments described in this paper. In addition, we compared mice who underwent primary tumor resection to mice who underwent a sham operation, not just mice that did not undergo a second surgery, to exclude bias due to missing surgical trauma in the control group.

However, our study also has some limitations. Unlike Di Trapani et al. [28], we did not perform complete resection of the prostate but only removed its tumor-bearing parts. The fact that leaving the normal mouse prostate in place affects the disease course compared to complete resection of the prostate can be doubted but not excluded. However, such a procedure would only be feasible when using a urinary diversion, as described by Di Trapani et al., which cannot be applied in immunodeficient mice. Even though we are experienced in the use of urological cancer mouse models, resection of a primary tumor induced after intraprostatic PCa cell inoculation proved to be highly technically demanding. During model establishment, we refined and optimized our perioperative analgesia and fluid resuscitation schedules as well as the methods applied for tumor resection and hemostatic control. However, the perioperative mortality rate was still around 50% after the second surgery and significantly higher in the primary tumor resection group compared with the sham operation group, which clearly impairs the conclusions to be drawn from the further course of the surviving animals. Of note, mice that died prematurely and were autopsied did not show any signs of peritonitis or bleeding as a putative cause of death. These findings show that it is primarily the general trauma and stress associated with two major abdominal surgeries within 8 weeks that impaired the survival of the mice in our study, which can only be managed by optimizing perioperative supportive care. Another point to consider is that our model does not adequately represent oligometastatic cancer, since at the moment of second surgery only lymph node metastases were detected and no bone metastases. Accordingly it could rather be regarded as an “M1a model”. In the future, it would be desirable to also have an “M1b model” available, i.e., a model with spontaneous development of bone metastases. Of note, the optimal time point for primary tumor resection, which was 8 weeks after orthotopic tumor cell injection in our study, was defined based on surgical feasibility and the presence of lymph node metastases. It is not clear if this time point truly represents the biological switch from oligometastatic to aggressive polymetastatic disease. Of course, our preclinical data have to be regarded as exploratory and direct translation to the human situation is limited by the above-discussed points. Furthermore, in future studies it would be desirable to used one or more additional PCa cell lines, since this would better reflect the molecular heterogeneity of prostate cancer.

After having overcome the above-described challenges, like reducing perioperative mortality, and implementing bioluminescence imaging and further cell lines, this innovative model will enable to answer several important scientific questions: Does removal of the primary tumor have an effect on the response rate and response duration of subsequent systemic therapies in metastatic prostate cancer, and, if yes, what are the underlying molecular mechanisms [13]? Here, the experiments in this study woul be performed with mice being castrated and/or receiving androgen deprivation therapy. How does the primary tumor contribute to the formation of premetastatic niches? How do extracellular vesicles and other mediators secreted by the primary tumor affect the biological behavior of metastases? Does the proliferation rate, gene expression, and/or miRNA expression in metastases change upon primary tumor removal?

## 5. Conclusions

We established an orthotopic mouse model of cytoreductive primary tumor resection in oligometastatic PCa and demonstrated the beneficial effects of such an intervention on disease progression. This model provides a valuable and versatile tool for further unraveling the complex molecular mechanisms involved in the disease of oligometastatic PCa, a clinical scenario whose optimal management will be increasingly demanding in the future. Special areas of interest in such a model include, among others, the mechanisms underlying premetastatic niche formation; the involvement of potential mediators of the primary tumor–metastases interaction, such as extracellular vesicles [31,32]; and the effect of the primary tumor on the response to systemic treatment and resistance development of metastases [13].

## Figures and Tables

**Table 1:** Defining the optimal time point for cytoreductive primary tumor resection. Mice with orthotopic tumors were analyzed for the presence of lymph node metastases (*n* = 3 per time point) and the resectability of the primary tumor (*n* = 2 per time point).

Time after Tumor Cell Implantation	6 Weeks	8 Weeks	10 Weeks
**primary tumor resectable**	yes	yes	No
**presence of lymph node metastases**	2/3 mice	3/3 mice	3/3 mice

## Data Availability

I would like to exclude this statement as all data are reported in this article.

## References

[1] Chaloupka M., Herlemann A., Spek A., Gratzke C., Stief C. (2017). Die zytoreduktive radikale Prostatektomie beim metastasierten Prostatakarzinom Cytoreductive, radical prostatectomy in metastatic prostate cancer. Der Urol..

[2] Mathieu R., Korn S.M., Bensalah K., Kramer G., Shariat S.F. (2017). Cytoreductive radical prostatectomy in metastatic prostate cancer: Does it really make sense?. World J. Urol..

[3] Heng D.Y., Wells J.C., Rini B.I., Beuselinck B., Lee J.-L., Knox J.J., Bjarnason G.A., Pal S.K., Kollmannsberger C.K., Yuasa T. (2014). Cytoreductive Nephrectomy in Patients with Synchronous Metastases from Renal Cell Carcinoma: Results from the International Metastatic Renal Cell Carcinoma Database Consortium. Eur. Urol..

[4] Choueiri T.K., Xie W., Kollmannsberger C., North S., Knox J.J., Lampard J.G., McDermott D.F., Rini B.I., Heng D.Y. (2011). The Impact of Cytoreductive Nephrectomy on Survival of Patients With Metastatic Renal Cell Carcinoma Receiving Vascular Endothelial Growth Factor Targeted Therapy. J. Urol..

[5] Bristow R.E., Tomacruz R.S., Armstrong D.K., Trimble E.L., Montz F.J. (2002). Survival effect of maximal cytoreductive surgery for advanced ovarian carcinoma during the platinum era: A meta-analysis. J. Clin. Oncol..

[6] Gundem G., Van Loo P., Kremeyer B., Alexandrov L.B., Tubio J.M., Papaemmanuil E., Brewer D.S., Kallio H.M., Högnäs G., Annala M. (2015). The evolutionary history of lethal metastatic prostate cancer. Nature.

[7] Kaplan R.N., Rafii S., Lyden D. (2006). Preparing the “soil”: The premetastatic niche. Cancer Res..

[8] Haffner M.C., Zwart W., Roudier M.P., True L.D., Nelson W.G., Epstein J.I., De Marzo A.M., Nelson P.S., Yegnasubramanian S. (2021). Genomic and phenotypic heterogeneity in prostate cancer. Nat. Rev. Urol..

[9] Culp S.H., Schellhammer P.F., Williams M.B. (2014). Might Men Diagnosed with Metastatic Prostate Cancer Benefit from Definitive Treatment of the Primary Tumor? A SEER-Based Study. Eur. Urol..

[10] Swanson G., Thompson I., Basler J., Crawford E.D. (2006). Metastatic Prostate Cancer—Does Treatment of the Primary Tumor Matter?. J. Urol..

[11] Heidenreich A., Pfister D. (2020). Radical cytoreductive prostatectomy in men with prostate cancer and oligometastatic disease. Curr. Opin. Urol..

[12] Parker C.C., James N.D., Brawley C.D., Clarke N.W., Hoyle A.P., Ali A., Ritchie A.W.S., Attard G., Chowdhury S., Cross W. (2018). Radiotherapy to the primary tumour for newly diagnosed, metastatic prostate cancer (STAMPEDE): A randomised controlled phase 3 trial. Lancet.

[13] Niklas C., Saar M., Nini A., Linxweiler J., Siemer S., Junker K., Stoeckle M. (2021). Can local treatment prolong the sensitivity of metastatic prostate cancer to androgen deprivation or even prevent castration resistance?. World J. Urol..

[14] Hajili T., Ohlmann C.H., Linxweiler J., Niklas C., Janssen M., Siemer S., Stoeckle M., Saar M. (2018). Radical prostatectomy in T4 prostate cancer after inductive androgen deprivation: Results of a single-institution series with long-term follow-up. BJU Int..

[15] Rexer H. (2015). Metastatic, hormone-naive prostate cancer interventional study: Multicenter, prospective, randomized study to evaluate the effect of standard drug therapy with or without radical prostatectomy in patients with limited bone metastasized prostate cancer (G-RAMPP—The AUO AP 75/13 study). Urol. A.

[16] Steuber T., Berg K.D., Røder M.A., Brasso K., Iversen P., Huland H., Tiebel A., Schlomm T., Haese A., Salomon G. (2017). Does Cytoreductive Prostatectomy Really Have an Impact on Prognosis in Prostate Cancer Patients with Low-volume Bone Metastasis? Results from a Prospective Case-Control Study. Eur. Urol. Focus.

[17] Poelaert F., Verbaeys C., Rappe B., Kimpe B., Billiet I., Plancke H., Decaestecker K., Fonteyne V., Buelens S., Lumen N. (2017). Cytoreductive Prostatectomy for Metastatic Prostate Cancer: First Lessons Learned From the Multicentric Prospective Local Treatment of Metastatic Prostate Cancer (LoMP) Trial. Urology.

[18] Hofman M.S., Lawrentschuk N., Francis R., Tang C., Vela I., Thomas P., Rutherford N., Martin J.M., Frydenberg M., Shakher R. (2020). Prostate-specific membrane antigen PET-CT in patients with high-risk prostate cancer before curative-intent surgery or radiotherapy (proPSMA): A prospective, randomised, multicentre study. Lancet.

[19] Connor M.J., Dubash S., Bass E.J., Tam H., Barwick T., Khoo V., Winkler M., Ahmed H.U. (2020). Clinical Translation of Positive Metastases Identified on Prostate-specific Membrane Antigen Positron Emission Tomography/Computed Tomography Imaging in the Management of De Novo Synchronous Oligometastatic Prostate Cancer. Eur. Urol. Focus.

[20] Saar M., Körbel C., Linxweiler J., Jung V., Kamradt J., Hasenfus A., Stöckle M., Unteregger G., Menger M.D. (2015). Orthotopic tumorgrafts in nude mice: A new method to study human prostate cancer. Prostate.

[21] Valta M.P., Zhao H., Saar M., Tuomela J., Nolley R., Linxweiler J., Sandholm J., Lehtimäki J., Härkönen P., Coleman I. (2016). Spheroid culture of LuCaP 136 patient-derived xenograft enables versatile preclinical models of prostate cancer. Clin. Exp. Metastasis.

[22] Linxweiler J., Körbel C., Müller A., Hammer M., Veith C., Bohle R.M., Stöckle M., Junker K., Menger M.D., Saar M. (2018). A novel mouse model of human prostate cancer to study intraprostatic tumor growth and the development of lymph node metastases. Prostate.

[23] Linxweiler J., Hajili T., Körbel C., Berchem C., Zeuschner P., Müller A., Stöckle M., Menger M.D., Junker K., Saar M. (2020). Cancer-associated fibroblasts stimulate primary tumor growth and metastatic spread in an orthotopic prostate cancer xenograft model. Sci. Rep..

[24] Young S.R., Saar M., Santos J., Nguyen H.M., Vessella R.L., Peehl D.M. (2013). Establishment and serial passage of cell cultures derived from LuCaP xenografts. Prostate.

[25] Nguyen H.M., Vessella R.L., Morrissey C., Brown L.G., Coleman I.M., Higano C.S., Mostaghel E.A., Zhang X., True L.D., Lam H.-M. (2017). LuCaP Prostate Cancer Patient-Derived Xenografts Reflect the Molecular Heterogeneity of Advanced Disease and Serve as Models for Evaluating Cancer Therapeutics. Prostate.

[26] Van den Broeck W., Derore A., Simoens P. (2006). Anatomy and nomenclature of murine lymph nodes: Descriptive study and nomenclatory standardization in BALB/cAnNCrl mice. J. Immunol. Methods.

[27] Cifuentes F.F., Valenzuela R.H., Contreras H.R., Castellón E.A. (2015). Surgical cytoreduction of the primary tumor reduces metastatic progression in a mouse model of prostate cancer. Oncol. Rep..

[28] Di Trapani E., Nini A., Locatelli I., Buono R., Russo A., Dell’Oglio P., Castiglione F., La Croce G., Benigni F., Montorsi F. (2018). Development of the First Model of Radical Prostatectomy in the Mouse: A Feasibility Study. Eur. Urol..

[29] Greenberg N.M., DeMayo F., Finegold M.J., Medina D., Tilley W.D., Aspinall J.O., Cunha G.R., Donjacour A.A., Matusik R.J., Rosen J.M. (1995). Prostate cancer in a transgenic mouse. Proc. Natl. Acad. Sci. USA.

[30] Tai S., Sun Y., Squires J.M., Zhang H., Oh W.K., Liang C.-Z., Huang J. (2011). PC3 is a cell line characteristic of prostatic small cell carcinoma. Prostate.

[31] Wortzel I., Dror S., Kenific C.M., Lyden D. (2019). Exosome-Mediated Metastasis: Communication from a Distance. Dev. Cell.

[32] Linxweiler J., Junker K. (2020). Extracellular vesicles in urological malignancies: An update. Nat. Rev. Urol..

